# Understanding the role of ageing in the thermal responses of life history and fitness in *Daphnia magna*

**DOI:** 10.1098/rspb.2025.0430

**Published:** 2025-07-23

**Authors:** Yngvild Vindenes, Catharina Broch, Tom Andersen, Dag O. Hessen, Torbjørn Ergon

**Affiliations:** ^1^ Centre for Ecological and Evolutionary Synthesis, University of Oslo, Oslo, Norway; ^2^ Section for Aquatic Biology and Toxicology, Department of Biosciences, University of Oslo, Oslo, Norway

**Keywords:** longevity, life history, poikilotherms, senescence, phenotypic plasticity

## Abstract

The current climate warming has raised concerns that increased rates of ageing will lead to reduced fitness and population viability in ectotherms. However, it is currently not known whether temperature effects on the rate of ageing mainly reflect a change in pace of life or changes in the strength of ageing in the life history. Evaluating this question requires that ageing in different temperatures is compared on the same intrinsic pace-standardized time scale. We present results from a laboratory experiment with *Daphnia magna*, recording complete life histories of 240 individuals from four clonal lines at six temperatures (5°C to 30°C). For each clone and temperature, we calculated measures of pace and shape of ageing in survival and reproduction. Fitness (long-term population growth rate) and mean lifetime reproduction were calculated using matrix population models. Our results highlight three main points: (i) lifespan is not a good predictor of temperature effects on ageing; (ii) there is no strong consistent effect of temperature on shape of ageing; and (iii) within temperature, the shape of ageing was positively correlated with fitness. Hence, lower fitness and population viability of ectotherms in warmer environments may be driven by factors other than increased ageing rates.

## 1. Introduction

Biological ageing refers to the decline in an adult organism’s functioning over time due to complex interactions of internal processes [1,2]. In the mean life history of a species or population, ageing is defined as increased mortality and/or decreased reproduction in later adulthood, with significant variability among species and individuals [3]. Some species also show ’negative ageing’, where the risk of mortality declines and the reproductive rate increases in late life [3]. For ectotherms, concerns have been raised that climate warming could potentially reduce population viability through accelerated ageing [4,5]. Temperature is known to have a strong direct effect on most biochemical and physiological rates of ectotherms [6–10], so increased rates of ageing with temperature are expected. However, if an organism simply completes the entire life cycle faster, ageing at a faster rate but also reproducing at a faster rate, the total amount of ageing in the life history would not be affected. On the other hand, if the shape of the change in mortality and reproduction through life is altered, the amount of ageing in the life history would also be affected [11]. We currently have limited understanding of how temperature affects ageing in the life history of ectotherms, and how this may affect mean lifespan and population viability.

To compare the amount of ageing in life histories for species and populations with different mean lifespans, Baudisch [11] suggested distinguishing between the ‘pace of life’ and ‘shape of ageing’. Measures of pace, such as mean lifespan, are time-dependent and reflect how fast life typically progresses for the organism [11,12]. Shape measures are dimensionless and reflect how much the mortality hazard or the reproductive rate changes through life, and in what direction [11,13,14]. In other words, shape measures the overall strength of ageing in a life history. Two populations with different rates of ageing may still show the same shape of ageing when measured on a pace-standardized scale, or even opposite patterns for the shape of ageing. Interspecific comparisons have shown, for example, that long-lived species can have larger shapes of ageing than short-lived species, and thus stronger ageing in their life history, although their rate of ageing is slower [11,13]. By standardizing the mortality hazard rate and reproductive rate to an intrinsic time scale of pace-standardized age, the shape of the changes in mortality or reproduction can be directly compared for different life histories [11,13].

Studies of the effects of climate warming on ectotherms often focus on changes in lifespan [15]. However, a reduced lifespan is not necessarily caused by an increased rate of ageing, as it can also result from an increase in the mortality hazard rate at all ages (i.e. age-independent mortality [16,17]). Since temperature affects most biological rates for ectotherms, the baseline expectation is that it also affects the rate of ageing [5]. However, temperature may also affect the age-independent mortality level, for example, through age-independent stress responses, which also affect lifespan. Although some studies have separated the effects of age-independent and age-dependent mortality [5], the degree to which lifespan correlates with the rate of ageing in ectotherms is not well known. Fitness is often measured as the long-term population growth rate λ, which is a function of the demographic rates across the entire life cycle [18]. While λ is a time-dependent rate (measuring population growth per time step), the mean lifetime reproduction $R_{0}$ represents population growth per generation. To compare the performance of life histories at different temperatures, including the role of ageing, $R_{0}$ may be most relevant as it is a measure of performance on an intrinsic time scale (generation time) for population growth. Thermal performance curves are often used to describe temperature responses of various performance measures in ectotherms [10,19]. The width and shape of a thermal performance curve reflect thermal plasticity, where generalists have wider thermal ranges but lower optima than specialists [10]. The performance curves of λ and $R_{0}$ are expected to show different shapes, with λ being maximized at a higher temperature than $R_{0}$ [20]. While most ectotherms show physiological and behavioural plasticity, allowing them to live in environments with thermal variability, the ongoing climate change may push more ectotherms out of their historic thermal range towards ranges of stressful temperatures [4].

Both extremes of the thermal range of an ectothermic organism can be considered stressful, but in different ways. At high temperatures, increased oxidative stress may cause accumulation of DNA damage, destabilization of cell regulation and reduced function of proteins and membranes, all potentially contributing to increased rates of ageing and earlier onset of ageing [4,21,22]. At low temperatures, reduced enzyme function and disrupted cell membrane potentials due to altered physical properties of lipids can limit the nutrient uptake even in a high-resource environment, and thereby limit the growth and development [15]. Ectotherms have developed various acclimation mechanisms to mitigate these negative effects induced by stressful cold or warm temperatures. For example, they may alter the lipid composition and protein structures in cold environments or upregulate heat shock proteins in warm environments [15]. However, these mechanisms can also be costly and may divert resources away from other processes such as development, reproduction and growth.

In this study, we explore the effects of temperature on the pace and shape of ageing in both survival and reproductive rate in a laboratory experiment of *Daphnia magna*. With many advantageous properties, such as clonal reproduction, combined with their importance in freshwater ecosystems, *Daphnia* has been used as a model organism in ecology and evolution for more than a century [23,24]. We calculate shapes of ageing in survival and reproductive rate (the latter also depends on the moulting rate) to measure the amount of ageing in the life history, using mean lifespan as a measure of pace of life. To compare these results with effects of temperature on fitness λ and net reproductive rate $R_{0}$, we construct matrix population models for each combination of clone and temperature [18].

## 2. Material and methods

### (a) Study species and clonal lines

*D. magna* is a freshwater crustacean with a wide geographic distribution [25]. It is typically found in small fish-free ponds and shows a high degree of phenotypic plasticity as an adaptation to varying food conditions [26] and abiotic conditions such as temperature [27,28]. Throughout life, individuals undergo regular moulting events [25], where moulting intervals are strongly related to temperature within the juvenile and adult phases [29]. Other life history processes are linked to the moulting process, including developmental stages and maturation in the juvenile phase and reproduction in the adult phase [25]. The clutch size typically increases throughout the first reproductive events [30,31]. We used four clonal lines (genotypes, here referred to as clones) for the experiment, brought to our university from the University of Basel: MA-ES (Morocco), IT-MDV (Italy), SE-G1 (Sweden) and SE-BY (Sweden). Detailed information is provided in electronic supplementary material, appendix S4.

### (b) Experimental design

The experimental design is described in detail in appendix S4, including information on stock culturing and preparation of the experimental animals. The experiment was conducted at the University of Oslo in 2017 and 2018, starting with 10 individuals per clone at each of six constant temperature treatments: 5°C, 10°C, 15°C, 20°C, 25°C and 30°C (in total 240 individuals, kept separately in 100 ml jars). The highest and lowest temperatures are close to the limits of the thermal range for the active life stages of the species [32,33]. Individuals were given regular changes of medium (ADaM [34]) and kept under *ad libitum* food conditions, at a stable 16:8 h light:dark cycle. The main food source was live *Chlamydomonas reinhardtii* algae, which were grown in continuous cultures in WC medium [35], supplemented by small amounts of frozen *Nannochloropsis* (see details on feeding protocol in electronic supplementary material, appendix S4).

Individual data on survival, moulting (when a carapace is shed), maturation (when eggs are visible in the brood pouch for the first time) and reproduction (number of neonates released) were recorded each day by visual inspection. Individuals were also filmed repeatedly to obtain size estimates using a non-invasive approach [36]. The filming procedure is described in electronic supplementary material, appendix S5, along with supplementary analyses of juvenile and adult growth and effects of body length on clutch size.

### (c) Statistical models

Statistical models were fitted to the data for moulting, survival, maturation and clutch size with respect to age or stage (overview in table 1). All analyses were done with the software R v 4.5.1 [37]. Detailed presentations of the statistical methods and model outputs, including R code, are given in electronic supplementary material, appendix S1; only a short summary is presented here.

**Table 1.** Overview of statistical models fitted to the individual life history data (details provided in electronic supplementary material, appendix S1). The standard deviation of residuals depends on temperature in some models, indicated by σ∼ temp in the model structure column. Random effects of individual ID were modelled on the intercept for all models with repeated individual measurements. The binomial models were fitted with a complementary log-log link function for the probabilities (indicated in the column 'linear predictor', empty cells in this column mean the link function is the identity function). $p_{\text{moult}}$: daily probability of moulting; $p_{\text{death}}$: daily probability of dying; $p_{\text{mature}}$: daily probability of maturing; ageLM: age at last moult; TLM: time since last moult; phase: juvenile or adult; stage: stage depending on previous moults within phase; age: age in days.

response	linear predictor	dataset	model structure of fixed effects (R syntax)	dist.
moult	ln(−ln(1 − $p_{\text{moult}}$))	all	clone × temp + temp × phase × ageLM + ln(TLM) × phase + ln(TLM) × temp	binomial
moult	ln(−ln(1 − $p_{\text{moult}}$))	juveniles	clone × stage + temp × stage + ln(TLM)	binomial
moult	ln(−ln(1 − $p_{\text{moult}}$))	adults	clone × stage + temp × stage + temp × ln(TLM)	binomial
death	ln(−ln(1 − $p_{\text{death}}$))	all	clone × age + temp × age + clone × temp	binomial
death	ln(−ln(1 − $p_{\text{death}}$))	all	clone × temp + clone × ageLM + temp × ageLM + temp × TLM	binomial
age		juveniles	clone × temp + stage × temp + I(stage2) + stage × clone, σ ∼ temp	normal
age		adults	stage × temp + I(stage2) + stage × clone, σ ∼ temp	normal
ageLM		juveniles	clone × temp + stage × temp +I(stage2) + stage × clone, σ ∼ temp	normal
ageLM		adults	stage × temp + I(stage2) + stage × clone, σ ∼ temp	normal
maturation	ln(−ln(1 − $p_{\text{mature}}$))	juveniles	stage × temp + clone	binomial
clutch size		adults	age × temp + age × clone + I(age2) × temp, σ ∼ temp	normal
clutch size		adults	stage × temp + stage × clone + I(stage2) × temp, σ ∼ temp	normal

All models included temperature and clone as categorical predictor variables, and either age, stage (within juvenile and adult phase), or age at last moult and time since last moult among the predictors (table 1). To estimate stage-specific survival, we used a separate model to estimate age based on stage, which was used to convert age-specific to stage-specific survival. The fitted mortality models correspond to a Gompertz mortality hazard rate with respect to age, while the moulting models correspond to a Weibull hazard function with respect to time since last moult. The latter was used to estimate intermoult intervals (electronic supplementary material, appendix S1).

### (d) Pace and shape of ageing

We define parametric measures of pace of life and shape of ageing in survival based on the Gompertz mortality hazard calculated from the fitted mortality model. For a given clone and temperature, the mortality hazard for age t can be written as $h(t)=b_{S}\eta e^{b_{S}t}$ (log hazard $\ln\;h(t)=\ln(b_{S}\eta )+b_{S}t$), where $b_{S}>0$ is the rate of increase in log mortality hazard with age, while η>0 is a shape parameter (not to be confused with shape of ageing). The age-independent component of the hazard rate corresponds to $b_{S}\eta$ while $e^{b_{S}t}$ is the age-dependent component. The mean lifespan L is our measure of pace, which in the Gompertz distribution is given by


(2.1) $$\begin{array}{lrr} & L=\frac{1}{b_{S}}e^{\eta }\text{Ei}(-\eta ), & \end{array}$$

where Ei(−η) denotes the exponential integral $\text{Ei}(-\eta )=\int_{\eta }^{\infty }(e^{-v}/v)dv$. The pace-standardized mortality hazard rate is then given by [11]


(2.2) $$\begin{array}{lrr} & h^{*}(t/L)=(b_{S}\eta )Le^{b_{S}t}=h(t)L. & \end{array}$$

This corresponds to dividing the hazard rate by the mean mortality hazard rate [11]. The standardized hazard rate allows a direct comparison of when in life (as measured in units of mean lifespans) the mortality hazard increases beyond the average, i.e. when $h^{*}(t/L)=1$.

The shape of ageing in survival ($S_{1}$) is the relative increase in hazard rate over one lifespan [11], i.e.


(2.3) $$\begin{array}{lrr} & S_{1}=\frac{h(L)}{h(0)}=\exp(Lb_{S})=\exp(e^{\eta }\text{Ei}(-\eta )). & \end{array}$$

Hence, $S_{1}$ is a transformation of the Gompertz shape parameter η. Since η>0 the shape is restricted to $S_{1}>1$, where values close to 1 indicate weak or no ageing, and larger values correspond to stronger ageing in the life history.

For the reproductive rate r(t), we base our shape estimate on the decline after peak reproduction, when measured on an intrinsic timescale of mean lifespan (pace L). We chose to focus on the decline phase since *Daphnia* typically shows a peak in reproduction [38,39]. The reproductive rate was defined as clutch size divided by the predicted intermoult interval for the relevant moulting event (electronic supplementary material, appendix S2). The age at peak reproductive rate for each clone and temperature were set manually through visual inspection (electronic supplementary material, figure S27). A separate log-linear model was fitted to the reproductive rate after the peak within each clone and temperature, i.e. $r(t)=a_{R}\exp\;b_{R}t$, where $a_{R}>0$ defines the intercept and $b_{R}$ is the slope of the log-reproductive rate. The pace-standardized reproductive rate is then


(2.4) $$\begin{array}{lrr} & r^{*}(t/L)=a_{R}L\;\exp(b_{R}t)=r(t)L. & \end{array}$$

We define the shape of ageing in reproduction as the relative decline in the reproductive rate over one lifespan,


(2.5) $$\begin{array}{lrr} & S_{2}=\frac{r(0)}{r(L)}=\exp(-b_{R}L). & \end{array}$$

Values of $S_{2}>1$ correspond to positive ageing, which is the only option we consider here since we only estimated the shape for clones and temperature where a clear peak was visible (for the rest, there is no support for ageing; see details in electronic supplementary material, appendix S2). Larger values of $S_{2}$ indicate stronger ageing.

### (e) Matrix population models

To calculate fitness λ (mean population growth per day), mean lifetime reproduction $R_{0}$ (mean population growth per generation) and generation time, we defined matrix population models for each clone and temperature, using parameter values from the statistical models. The matrix models represent density-independent dynamics, as in the early growth phase of a newly established population. We used standard methods for matrix population models to estimate the population parameters [18], and a detailed description of the model construction and parameters is provided in electronic supplementary material, appendix S3.

While λ is sensitive to the timing of life-history events, $R_{0}$ is not [18]. Thus, we expect that λ will be highly affected by temperature through its effects on the timing of moulting, maturation and reproduction. In contrast, $R_{0}$ represents the mean lifetime reproduction irrespective of when the offspring are produced, so we expect this parameter to be affected by temperature through effects on stage-specific survival and clutch size. We consider $R_{0}$ as a suitable measure in this study to compare the performance of one clone at different temperatures [20], while λ is relevant measure for comparing the fitness of different clones within each temperature.

## 3. Results

### (a) Age-specific temperature responses

**Figure 1.**
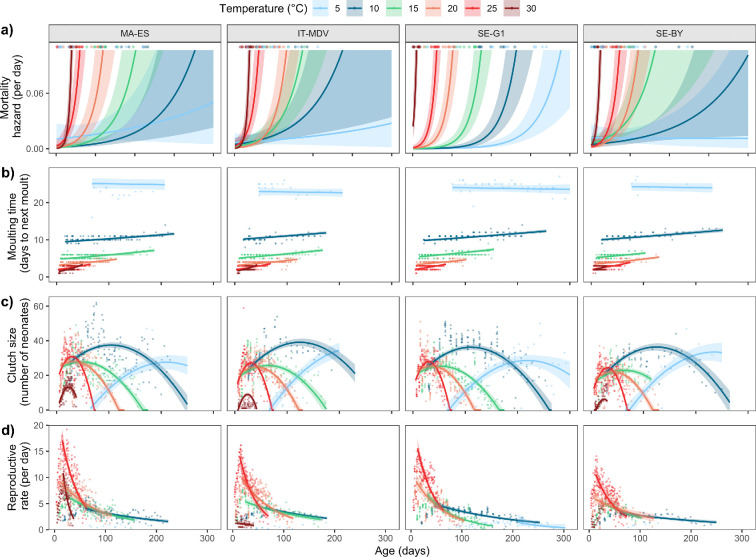
Age-specific temperature responses of (a) Gompertz mortality hazard rate from binomial mortality model, (b) Moulting time predicted from the binomial moulting model (adults only, for juveniles, see electronic supplementary material, figures S2 and S3), (c) Clutch size predicted from a quadratic model, and (d) Predicted reproductive rate (clutch size divided by predicted moulting time), with log-linear models fitted to the decline phase (see details in text). Lines are model predictions, shaded ribbons 95% confidence intervals, points represent individual data. Each panel represents results for one clone (clone identity of the panels follows the same order as in (a). Electronic supplementary material, figure S31 shows the same figure with panels organized by temperature.

The *Daphnia* showed substantial variation in mean lifespan, from (considering groups that reproduced) 23 days for the SE-BY clone at 30°C to 254 days for the SE-G1 clone at 5°C (table 2). The longest-living individual (340 days) was from the SE-G1 clone at 5°C. The mortality hazard rates predicted from the binomial model (figure 1a; electronic supplementary material, figure S8) correspond well with the temperature and clone patterns of the non-parametric Kaplan–Meier curves (electronic supplementary material, figure S7). Temperature had a strong positive effect on the rate of ageing in survival, as measured by the slope of the log-hazard rate (electronic supplementary material, figure S9). However, the effects on the initial hazard rate (age-independent mortality) varied among the clones, reflecting large differences in temperature response of early survival (figure 1; electronic supplementary material, figures S7–S9). The temperature responses of the SE-G1 clone stood out from the others, as it showed a higher survival at lower temperatures and very low survival at the highest temperature (table 2; electronic supplementary material, figure S7).

**Table 2.** Summary statistics for each clone and temperature (°C) group. Mean and range (in brackets) for age at maturation (AgeMat, days), number of moults at maturation (stageMat), age at death (ageDeath, days), number of completed stages and lifetime reproductive output (LRO, total number of neonates produced). nMat is the number of individuals that matured, each group started with 10 individuals. Statistics for age/stage at death and for LRO were calculated after removing data for six individuals who were killed by accident. For median values, see electronic supplementary material, appendix S1, table S2.

temp	clone	nMat	ageMat	stageMat	ageDeath	stages	LRO
5	MA-ES	3	69.7 (68, 72)	7 (7,7)	67.9 (7, 221)	4.2 (0, 13)	25.8 (0, 108)
5	IT-MDV	9	49.3 (46, 58)	5.2 (5,6)	93.7 (33, 162)	7 [3,12)	6.8 (0, 30)
5	SE-G1	9	94.4 (75, 109)	8.1 (7,9)	254.3 (116, 340)	12.8 (3, 17)	132.6 (19, 215)
5	SE-BY	5	85.2 (77, 89)	7.8 (7,8)	86.3 (2, 246)	6 (0, 14)	24.4 (0, 192)
10	MA-ES	10	19.6 (16, 21)	4.9 (4,5)	165.3 (113, 233)	18.2 (13, 24)	434.8 (291, 625)
10	IT-MDV	10	17.6 (17, 20)	4.1 (4,5)	92 (30, 185)	10.6 (5, 19)	223.2 (18, 544)
10	SE-G1	10	21 (21, 21)	5 (5,5)	193.6 (105, 260)	20.1 (12,26)	520.1 (294, 662)
10	SE-BY	6	20.8 (20, 21)	5 (5,5)	95.9 (2, 258)	10.2 (0, 25)	186.5 (0, 521)
15	MA-ES	10	9.5 (8,11)	4.7 (4,5)	114.8 (52, 196)	22.7 (11, 35)	433 (250, 573)
15	IT-MDV	10	9 (9,9)	4.1 (4,5)	76.3 (35, 178)	15.2 (8,31)	286.9 (115, 653)
15	SE-G1	10	12.1 (12,13)	5 (5,5)	133.5 (34, 161)	24 (8, 29)	395.2 (65, 487)
15	SE-BY	10	10.8 (10,14)	4.2 (4,5)	75.3 (24, 106)	14.7 (6,20)	222.6 (20, 333)
20	MA-ES	10	6 (5,7)	4.2 (4,5)	77.5 (35, 118)	19.4 (5,32)	466.4 (193, 697)
20	IT-MDV	10	6 (6,6)	4.1 (4,5)	96.1 (50, 125)	26.8 (15,33)	505.9 (268, 634)
20	SE-G1	10	7.8 (7,8)	5.1 (5,6)	78.3 (29, 107)	22.2 (11,29)	394.8 (175, 527)
20	SE-BY	10	6.6 (6,7)	4.1 (4,5)	70.4 (38, 138)	19.2 (12,33)	300.8 (178, 504)
25	MA-ES	7	4 (4,4)	4 (4,4)	38.9 (2, 66)	16.8 (1,28)	372.5 (0, 678)
25	IT-MDV	10	4.1 (4,5)	4.1 (4,5)	43.4 (20, 72)	18.2 (10,28)	344.6 (134, 546)
25	SE-G1	10	5.5 (5,6)	5.1 (5,6)	50 (15, 66)	21.1 (8,27)	387.1 (64, 529)
25	SE-BY	10	4.2 (4,6)	4.1 (4,5)	56.4 (19, 77)	22 (10, 28)	345.8 (100, 461)
30	MA-ES	10	5.1 (4,6)	5.1 (4,6)	38.6 (35, 52)	20.4 [18,26)	159.5 (135, 186)
30	IT-MDV	10	6.5 (6,7)	5.5 (5,6)	36.9 (15, 47)	18 (9, 23)	20.9 (8,40)
30	SE-G1	0			10.5 (1, 19)	4.6 (0, 10)	0 (0, 0)
30	SE-BY	10	5.6 (5,7)	5.3 (5,6)	23.1 (12,33)	11.7 (7,16)	31.7 (1, 72)

The predicted moulting probability from the binomial model increased with time since last moult, and decreased with age except for adults in the coldest treatment (electronic supplementary material, figures S1 and S4). The corresponding moulting times predicted from the binomial models were highly consistent between clones for each temperature (figure 1b; electronic supplementary material, figures S2 and S3) and increased with adult age except in the coldest treatment, suggesting that ageing is also causing a slowing down of this ‘moulting clock’ [40]. The moulting times also increased with age in the juvenile phase, but mainly in the three coldest treatments, and in particular, the coldest one (electronic supplementary material, figures S2a and S3a). The age at maturation was lowest at 25°C and highest in the coldest treatment (5°C), where many individuals also never reached maturation (table 2). At 30°C, none of the individuals in the SE-G1 clone survived to maturation.

Clutch sizes generally showed a classical pattern for *Daphnia*, with a peak in early adult life followed by a decline (figure 1c). Exceptions to this pattern were seen in the two extreme temperatures (5°C and 30°C), where most clones did not show any clear peak, due to poor survival and reproduction. The maximum clutch sizes reached were highest at 10°C and 5°C, similar at 15°C, 20°C and 25°C, and lowest at 30°C (the SE-G1 clone did not mature at this temperature). While body length had a positive effect on clutch size at the individual level, the difference in clutch size between temperatures could not be explained by body length, as individuals grew to similar lengths at all four non-extreme temperatures (electronic supplementary material, appendix S5). The maximum reproductive rates, measured as the predicted mean number of offspring per day, were found at 25°C, where the moulting rate is fast and the clutch size is still fairly large. There was a positive effect of temperature on the predicted rate of ageing in reproductive rate (rate of decline after the peak; figure 1d), however, at 30°C only one clone (MA-ES) showed a peak and strong decline, while the other clones showed either no reproduction (SE-G1) or no clear peak (no ageing; IT-MDV, SE-BY).

### (b) Standardized rates and pace and shape of ageing

**Figure 2.**
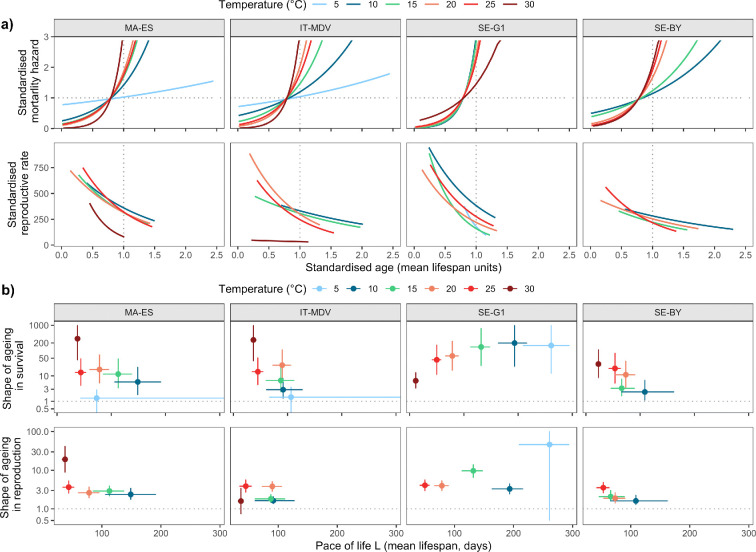
(a) Pace-standardized mortality hazard rate (first row) and reproductive rate (second row) against standardized age (units of mean lifespan), estimated from the fitted age-specific models (figure 1; see text for details). Note that the standardized mortality hazard for SE-G1 at 5°C is not visible because the line is hidden by other lines at intermediate temperatures. Other combinations of clone and temperature not shown in the figure represent cases with no evidence of ageing (see text). (b) Shape of ageing in survival (first row) and in reproductive rate (second row), shown on a log-transformed y-axis, by pace of life. Shape values close to 1 indicate no ageing, and larger values correspond to stronger ageing in the life history. Points are model predictions, lines are 95% confidence intervals (note that intervals for MA-ES, IT-MDV and SE-G1 at 5°C extend out of the panels, values are given in electronic supplementary material, table S3. Combinations of clone and temperature not shown in the figure have no evidence of ageing (see text). Electronic supplementary material, figure S33 shows the same figure with panels organized by temperature.

Overall, temperature had a negative effect on the mean lifespan predicted from the binomial models (table 2; figure 2; electronic supplementary material, figures S10, S11, S13–S16, S18 and S25). However, for two of the clones, the observed mean lifespan was lower at 5°C than at 10°C (table 2). At 5°C the estimated values of the Gompertz parameters $b_{S}$ and η were negative for the SE-BY clone (electronic supplementary material, figure S9), and for the two southern clones (MA-ES, IT-MDV) negative values occurred when simulating from the model. This resulted in infinite estimates for the mean lifespan L or its upper confidence limit for these three clones at 5°C and a negative infinite value of the shape or its lower limit (since the Gompertz distribution is only defined for $b_{S}>0$ and η>0). For these three clones, there is no evidence of ageing at 5°C, as indicated by the wide confidence intervals around the mortality hazard rate (figure 1a; electronic supplementary material, figure S25). Note that for SE-BY at 5°C the negative point estimates imply that the predicted hazard rate declines with age, and that the predicted cumulative survival in the model does not reach zero (although it reaches a value very close to zero; electronic supplementary material, figure S10).

The pace-standardized mortality hazard rate allows comparison of temperature responses on the same intrinsic time scale, measured in mean lifespans (figure 2a). Three clones (MA-ES, IT-MDV and SE-BY) showed a similar pattern where initial mortality declines with increasing temperature, being highest at 5°C and late-life mortality shows a stronger increase over standardized age. The fourth clone (SE-G1) showed a similar standardized hazard rate in all temperatures except the warmest, where initial mortality was high and the subsequent increase weaker through life (figure 2a). In all clones, there is a negative correlation between initial mortality and the amount of increase in mortality through life. The estimated shapes of ageing in survival reflect the shape of the standardized hazard rate (figure 2b; electronic supplementary material, figure S25), with the SE-G1 standing out from the other three. At the coldest temperature (5°C), only the SE-G1 clone had any ageing in survival.

The standardized reproductive rates (for the decline phase of reproduction) and the associated shape measures highlight the differences between temperatures on the common intrinsic time scale of lifespans (figure 2). The clones showed the most consistent responses at 25°C (figure 2; electronic supplementary material, figure S29). There was no evidence of ageing in reproductive rate for any of the clones at 5°C (figure 2; electronic supplementary material, table S4). The estimated decline in the reproductive rate on this intrinsic time scale is strong for the SE-G1 clone at this temperature; however, the confidence interval for the shape is wide and overlaps with 1 (figure 2b; electronic supplementary material, figure S29). At 30°C, only one clone (MA-ES) shows a strong decline in the standardized rate (figure 2b).

### (c) Stage-specific responses and matrix outputs

**Figure 3.**
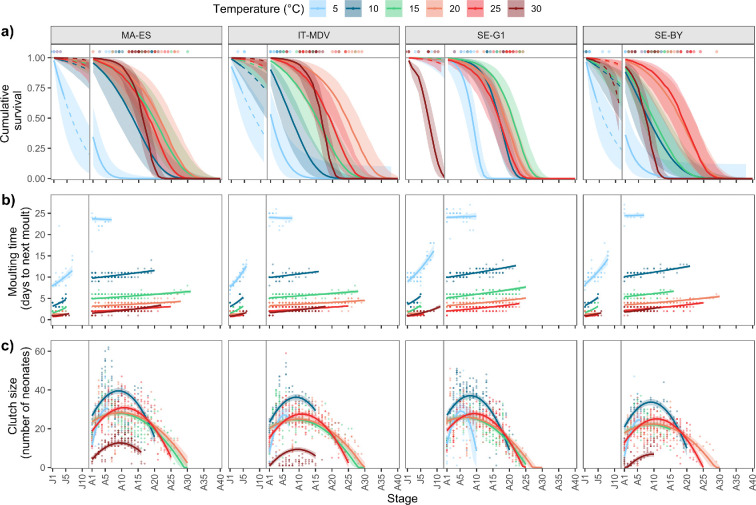
Stage-specific temperature responses of (a) cumulative survival, calculated from the binomial mortality model and translated from age to stage, (b) intermoult intervals based on binomial moulting models fitted separately to the juvenile and adult phase, and (c) clutch size with fitted quadratic models. Lines are predictions from fitted models, ribbons 95% confidence intervals, points represent individual data. Electronic supplementary material, figure S35 shows the same figure with panels organized by temperature.

Stage number is highly correlated with age within each temperature (electronic supplementary material, figure S12), but the relationship is not linear because the moulting intervals increase through life in both the juvenile and adult phase (figures 1 and 3; electronic supplementary material, figures S2 and S3). Therefore, we did not use stage as a measure of pace of life, but the stage-specific results highlight interesting differences between temperatures and were used to construct matrix models (figure 3). Individuals completed the highest number of stages and reproductive events (corresponding to adult stages) at intermediate temperatures of 15°C, 20°C and 25°C. At 10°C, clutch sizes were consistently larger and the number of reproductive events was lower than at higher intermediate temperatures (figure 3). At intermediate temperatures, individuals required a similar number of juvenile stages before maturation, and maturation was more synchronous within each clone (table 2). The peak in clutch size seems to occur at a relatively earlier stage in cold 5°C and 10°C,) versus warmer treatments; however, given the amount of variation in clutch size, this pattern is not certain (figure 3).

**Figure 4.**
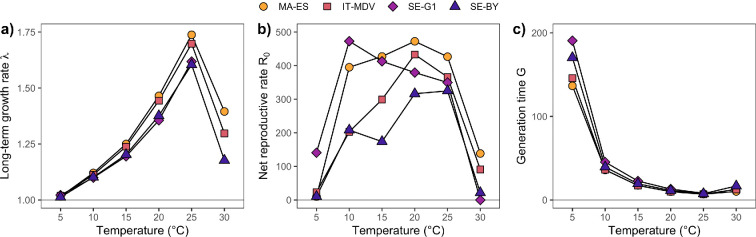
Outputs from matrix population models for each clone and temperature. (a) The long-term population growth rate λ (multiplicative daily increase). (b) Mean lifetime reproductive success $R_{0}$ (number of neonates produced). (c) Generation time (mean age of mothers in days). Individuals did not survive to maturation in the SE-G1 clone at 30°C, so that λ=0 (not shown) and $R_{0}=0$ for this combination, and generation time is not calculated.

The long-term growth rate λ (fitness) from the stage-structured matrix models shows a classical pattern of an ectotherm temperature response curve, with a maximum of 25°C for all clones (figure 4a). Although 30°C is considered a stressful temperature for this species, for the three clones that matured at this temperature, λ was still higher than 1 and comparable to or higher than the values in some lower temperatures (figure 4a). The clones showed very similar response curves of λ, with values somewhat lower for the two northern clones (SE-G1 and SE-BY) than the two southern ones (MA-ES and IT-MDV). Across most temperatures except the coldest, the two southern clones had the highest clutch sizes (electronic supplementary material, figure S21b).

The mean lifetime reproductive success $R_{0}$ was more variable between temperatures and clones than λ (figure 4b) and reflected mainly the clone differences in survival responses to temperature, given that clutch sizes showed similar responses. For SE-G1, the highest $R_{0}$ was found at 10°C, reflecting the high survival of this clone at low temperatures, while the three other clones had their highest $R_{0}$ values at 20°C or 25°C. All clones were able to maintain fairly high $R_{0}$ values in the four non-extreme temperatures. The generation time was much longer in the coldest temperature compared to the other treatments, and shortest at 25°C and largely reflects the responses of moulting time (figure 4c).

## 4. Discussion

The results of this study highlight important aspects regarding the role of ageing in ectotherm responses to temperature, with potential consequences for understanding ectotherm responses to global warming. First, we confirmed that lifespan is not a good predictor of the rate of ageing [16,17] because temperature can have different effects on the age-independent and age-dependent components of mortality. In all four clones, the slope of the log-mortality hazard increased consistently with temperature; however, the age-independent intercept showed different responses (electronic supplementary material, figure S9). As a result, mean lifespan was short for three of the clones at the coldest temperature, and there was considerable variation in mean lifespan also at other temperatures. Second, we found that temperature did not have strong or consistent effects on the strength of ageing in the life history. Third, the clones that showed the strongest ageing at a given temperature also had the highest fitness and mean lifetime reproduction among the clones at that temperature. This suggests that although climate warming may reduce the lifespan and population viability of ectotherms [4], these effects are not necessarily driven by increased ageing, but rather by early life effects of stress.

Lifespan is often used as a proxy for ageing in ectotherm responses to climate warming [4,15,21]. Some studies have warned against using mean lifespan as a proxy for ageing, for instance, with examples from comparative studies of mammals [17] and angiosperms [41]. The results of this study support such caution against interpreting changes in mean lifespan as ageing also in the context of ectotherm responses to warming, since temperature had a consistently strong effect on the rate of ageing in mortality, but variable effects on the age-independent mortality and thereby on lifespan.

The patterns of temperature effects on lifespan in this study are overall in line with other studies [8,42], although mortality levels seem to have been lower in this study compared to many others. Mortality is expected to be higher in natural conditions, in presence of competition, predation and parasites [25]. Thus, we expect that most individuals in natural *Daphnia* populations would die before ageing is manifested. A study of mosquitoes (*Anopheles gambiae* [43]) found that mortality levels were higher in wild populations than in lab populations. However, other studies on insects have demonstrated the presence of ageing also in natural populations [44]. Our study suggests that if ageing is observed in a natural *Daphnia* population, it could be a sign that the population is experiencing good growth conditions where many live to older ages, rather than a sign of stress.

While we studied responses to constant temperatures, natural populations also experience large fluctuations on a daily and seasonal basis. The results of this study reflect the large thermal plasticity of this species, which is one adaptation allowing it to live in highly variable environments [26,28]. Another adaptation to variability is switching to sexual reproduction to produce resting eggs that can survive long periods of harsh conditions and provide new genetic recombinations [25]. We did not record the sex of the neonates produced, but it is plausible that some were males. We observed one single ephippium throughout the entire experiment (in one of the SE-G1 clone individuals at 10°C). Since all experimental individuals were kept in separate jars under *ad libitum* conditions, they did not receive any other cues known to trigger production of males and ephippia (e.g. density and presence of males [25]).

Although the four clones originate from very different geographic locations, some of their temperature responses were remarkably similar, in particular the moulting rate, but also clutch size and fitness λ. The main difference between clones was seen in the survival responses, where one clone (SE-G1) stood out with higher survival at low temperatures and a low survival at the highest temperature. The survival differences lead to large differences in $R_{0}$. The similarity in moulting and clutch size responses suggests that these processes are controlled by genetically conserved processes, shared with other ecdysozoans. A meta-analysis based on 20 *Daphnia* species concluded that evolution of thermal plasticity is best understood at the population level [28]. We cannot conclude that the survival differences between clones represent local adaptation since we had only one clone per location; however, the differences we observed correspond with general expectations given their origin [33].

The temperature response of fitness λ, which is a time-dependent rate, reflects changes in the time-dependent rates of maturation, development and reproduction [20]. The time-independent mean lifetime reproduction $R_{0}$, on the other hand, is determined by clutch size, number of reproductive events and survival until each reproductive event. At the lowest temperature in this study, λ was mainly reduced by the long moulting time (affecting rates of development and reproduction), while $R_{0}$ was reduced by low early life survival and fewer reproductive events. At the highest temperature, both λ and $R_{0}$ were reduced by the low clutch sizes, but individuals still maintained a high moulting rate and (in three clones) high survival in early life. Hence, in the warmest temperature a stronger ageing of survival contributed to the decline in $R_{0}$ but to a smaller degree, the decline in λ. More research is needed to evaluate whether a positive link between the strength of ageing and fitness is common across ectotherms. The results from this study apply to a fast-growing organism with potentially many reproductive events. In species with large fluctuations in the population structure, where the population for some time period consists mainly of older adults (e.g. as seen in fluctuations in year class strength of fish [45]), increased ageing in warmer environments may still pose a threat to population viability. Semelparous species may also show different responses regarding strength of ageing in survival and its response to temperature, and more comparative research is needed across ectotherm species to evaluate the potential role of other life history characteristics and general ecology in determining the shape of ageing.

In general, longer development times at low temperatures imply a higher risk of mortality before maturation compared to higher temperatures, which could select for increased investment towards growth and early reproduction relative to somatic maintenance and, in turn, increased ageing according to the disposable soma theory [46]. We did see reduced survival in early life at low temperatures (for three of the four clones), along with larger clutch sizes. McKee & Ebert [31] also observed a negative effect of temperature on clutch size when food levels were high (comparing 12°C, 16°C and 22°C). However, we did not see any stronger ageing in late life in either survival or reproduction at low temperature. This suggests that the reduced survival in early life at low temperatures is not caused by reduced investment by individuals in somatic maintenance. Instead, it could be a result of limitations on nutrient uptake in cold conditions [15] and increased risks associated with the moulting process. Despite being in *ad libitum* food conditions, the individuals in this experiment may have had limited uptake and use of nutrients in the coldest treatments due to lower metabolism and reduced cell membrane function [15], as suggested by the observed increase in intermoult intervals with juvenile stage.

Life history trade-offs are often less visible under *ad libitum* food conditions [47,48]. Reducing food levels would probably affect the results of this study as in *Daphnia*, food level is often found to interact with temperature to determine life history responses [25,31,49]. Moderate calorie restriction has been shown to prolong the lifespan of many species, including *Daphnia* [50–53]. However, studying the combined effects of calorie restriction and temperature on ageing is complicated by the fact that specific nutritional demands also change with temperature [54]. For example, the effects of temperature on somatic growth are stronger at high compared to low food levels [49].

Few studies have measured the shape of ageing in reproduction, as most have focused on ageing in survival. The shape of ageing in reproduction of the *Daphnia* in this study largely showed a similar pattern to the shape of ageing in survival, although with smaller differences between temperatures. In general, defining a shape of ageing for reproduction is less straightforward than for survival, because for many organisms reproduction is not restricted to one event per individual [14]. Baudisch & Stott [14] used cumulative reproduction curves to define a shape measure, considering that if the total offspring number accumulates over age at a rate that decreases over age, this will lead to a concave cumulative reproduction curve, while an accelerating rate would lead to a convex curve representing negative ageing [14]. They then derived a measure for the shape of ageing reflecting the shape of this curve. This method is useful to characterize how reproduction changes through life in different iteroparous species. However, for life histories where the offspring accumulation first accelerates and then declines, the resulting cumulative curve will be convex in early life and concave in late life, and the corresponding shape measure would reflect both the increase phase and the decline phase. As our main aim was to characterize the strength of ageing in late life in an organism where the reproductive curve is known to typically have a peak, the decline phase is most relevant. We therefore derived a shape measure that is not influenced by early reproduction. This shape measure is also not sensitive to the length of the reproductive lifespan, which can be difficult to estimate reliably from data.

## 5. Conclusion

This study demonstrates how temperature can affect the pace and shape of ageing in an ectotherm. The results highlight that lifespan is not a reliable predictor of the effect of temperature on rate of ageing in ectotherms, and that stronger ageing in the life history may reflect high rather than low fitness. Global warming may lead to reduced mean lifespan and population viability in ectotherms, but our results suggest that increased rates of ageing may not be the main driver behind these responses. Instead, increased ageing may correlate with high fitness. More research is needed to evaluate the generality of these findings across other ectotherms with different types of life history and ecological roles.

## Data Availability

Electronic supplementary material, including code and data files, is available on Figshare [55].
